# Using Sentinel 2A and Landsat 8 imagery to assess changes in forest carbon storage

**DOI:** 10.1038/s41598-025-21607-0

**Published:** 2025-10-27

**Authors:** Bingjie Li, Shanghua Liu, Dongwei Liu, Zhitao Fan, Zhicheng Qu, Shunyu Yao, Xiashu Su, Lixin Wang

**Affiliations:** 1 School of Ecology and Environment, Inner Mongolia University, Hohhot, 010021 China; 2 Ordos Institute of Forestry and Grassland Science, Ordos, 017000 China; 3 Key Laboratory of River and Lake Ecology in Inner Mongolia Autonomous Region, Hohhot, 010021 China; 4 Key Laboratory of Ecology and Resource Use of the Mongolian Plateau, Ministry of Education of China, Hohhot, 010021 China

**Keywords:** Climate change, Forest carbon storage, Machine learning, Sentinel 2A, Landsat 8, Plant sciences, Ecology, Climate-change ecology, Environmental sciences, Environmental impact

## Abstract

Estimating carbon storage using high-resolution imagery of dominant species and types is often constrained by the availability of data. Herein, we developed a carbon storage estimation model for dominant species and types using high-resolution Sentinel 2A imagery and compared the two approaches using lower-resolution Landsat 8 imagery for whole-forest estimation. Approach 1 employs a traditional method using in-situ carbon storage measurements with Landsat 8 vegetation indices, whereas Approach 2 uses Sentinel 2A carbon storage estimates as a reference. Using Random Forest, Decision Tree, and Multiple Linear Regression models, we compared both approaches and found that Approach 2 estimates matched the Sentinel 2A results for different species and types more accurately, including *Populus*, *Salix*, *Pinus tabuliformis*, and shrub types. At the same time, our research results show that machine learning models effectively estimated carbon storage using Sentinel 2A imagery and dominant species classification. For the whole forest assessment with Landsat 8 imagery, Approach 2 yielded superior accuracy over Approach 1. This method enabled the calculation of historical carbon storage, showing that the Ordos Forest carbon storage increased by 27 Mt (89%) from 2013 to 2023, demonstrating the feasibility of long-term carbon monitoring using lower-resolution imagery.

**Supplementary Information:**

The online version contains supplementary material available at 10.1038/s41598-025-21607-0.

## Introduction

Current scientific research indicates that forest carbon storage continues to play a crucial role in the global carbon cycle. Forests convert carbon dioxide into biomass through photosynthesis, thereby regulating atmospheric CO_2_ concentrations and mitigating the greenhouse effect. Therefore, the accurate quantification of forest carbon storage is essential for understanding the global carbon budget, creating strategies to combat climate change, and implementing sustainable forest management practices. It is estimated that global forest ecosystems absorb approximately 3 billion tons of CO_2_ annually, equivalent to one-third of the global fossil fuel CO_2_ emissions ^1,2^. China is committed to reducing its carbon emissions and achieving carbon neutrality. The Chinese government has pledged to reach a peak in carbon emissions by 2030 and carbon neutrality by 2060. In recent years, China has enhanced its forest carbon sink capacity through forestry projects such as the Natural Forest Protection Project and the Grain for Green Program as an integral part of this commitment ^3,4^. At the global level, accurate assessment of forest carbon storage is essential not only for understanding the carbon cycle, but also for developing effective strategies to achieve carbon neutrality goals. This requires extensive research to better monitor, report, and verify changes in forest carbon storages ^5^ to establishing a scientific basis for future climate protection.

Traditionally, forest carbon storage has been estimated via field measurements of various tree parameters including species, height, diameter at breast height (DBH), and crown width in selected forest plots. These data were then used to calculate biomass using conversion factors or allometric growth equations. Carbon storage was subsequently calculated by determining the carbon content of the trees. Although this method provides high-precision data, it requires substantial labor and time, making it difficult to implement over large areas ^6–8^.

In recent years, remote-sensing technologies have been widely adopted to estimate large-scale forest carbon storage estimation ^9^. On a global scale, remote sensing provides an efficient, continuous, and comprehensive approach to estimate forest carbon storages ^10^. Simple linear regression models based on vegetation indices, particularly the Normalized Difference Vegetation Index (NDVI), are commonly used to estimate preliminary forest carbon storage estimates ^11^. However, these models have certain limitations: NDVI tends to saturate in areas with high biomass density, is sensitive to background soil reflectance in areas with sparse vegetation, and varies with seasonal changes and atmospheric conditions. Additionally, the relationship between the NDVI and carbon storage is often non-linear, especially in complex forest structures, making simple linear regression models inadequate for accurate estimation ^12,13^. These limitations significantly affect the reliability and precision of carbon storage estimates, particularly in areas with dense vegetation or heterogeneous forest structures. Consequently, nonparametric techniques have been introduced to invert the forest carbon storage. Machine learning models such as Random Forests (RF), Support Vector Machines (SVM), and Deep Neural Networks (DNNs) have become powerful tools for estimating forest carbon storage owing to their ability to process large volumes of non-linear data. These models enhance estimation accuracy and reliability by integrating multiple remote sensing data sources ^14,15^. Specifically, methods such as Random Forests and decision trees, which are known for their superior performance in handling complex datasets, have been widely applied to forest carbon storage estimation ^16,17^. Integrating traditional field measurements with advanced remote sensing techniques through data fusion significantly improves the precision of the estimates ^9,10^. Furthermore, the use of time-series remote-sensing data enables the monitoring of dynamic changes in forest carbon storage, which is crucial for assessing the impacts of climate change and human activities. Studies have demonstrated that the application of high-resolution remote-sensing data provides detailed information on forest cover and carbon storage dynamics, which are important for understanding the mechanisms of the global carbon cycle ^10,18^.

However, the application of high-resolution remote-sensing imagery for estimating carbon storage has been limited in recent years. This makes it difficult to track long-term changes in carbon storage over a long time ^19,20^. Ordos is an ideal case study area for examining forest carbon storage dynamics. As a typical arid-to-semi-arid ecological transition zone, it serves as a critical ecological barrier in China and has undergone extensive restoration through programs such as the Grain for Green Project. Its relatively simple vegetation structure, dominated by artificial forests and shrubs, reduces the complexity of carbon estimations typically found in diverse forest ecosystems. The region’s experience with concurrent economic development and ecological restoration makes it particularly representative of similar climate zones undergoing environmental rehabilitation. Thus, Ordos offers valuable insights for developing remote sensing-based carbon estimation methods for arid and semi-arid regions. Here, we used three models—Random Forest, Decision Tree and Multiple Linear Regression—to estimate the forest carbon storage of Ordos. Based on Sentinel 2A imagery, an optimal carbon storage estimation model for five dominant species and types of species in Ordos was established for 2023. The total carbon storage of the entire forest in Ordos was calculated by summing the carbon storage of these five dominant species and types species and types. Based on Landsat 8 imagery and the whole forest, we took two approaches: Approach 1 and Approach 2. In Approach 1, the estimates of carbon storage were calculated using traditional methods, specifically by directly modeling the measured carbon storage with vegetation indices from Landsat 8 imagery to estimate the carbon storage of the entire forest. In Approach 2, the estimates of carbon storage were first made based on Sentinel 2A imagery for each dominant species and type, which were then used as a reference for whole forest estimates based on Landsat 8 imagery. In this study, we categorized the scale of forest classification into two levels: dominant species and types and whole forests. The dominant species and types included *Populus*, *Salix*, P*inus tabuliformis*, other species and types, and shrubs. These categories were selected because Ordos is located in an arid to semi-arid region where the number and variety of trees are limited, whereas shrubs are abundant. Therefore, the carbon storage of shrubs is considered a significant part of the forest carbon storage in Ordos. A whole forest refers to an unclassified aggregation of all species and types. Landsat 8 estimates of carbon storage were made using Approaches 1 and 2 with the three models. The accuracy of the three models was analyzed, and the most accurate model was applied to Ordos. The Landsat 8 data, at a lower resolution, cover a much longer period than recent high-resolution satellite products. Our research design enables the alignment of carbon storage estimates based on low-resolution imagery with those based on high-resolution imagery, effectively overcoming the temporal limitations of high-resolution satellite products.

## Study area and data collection

### Study area

**Fig. 1:**
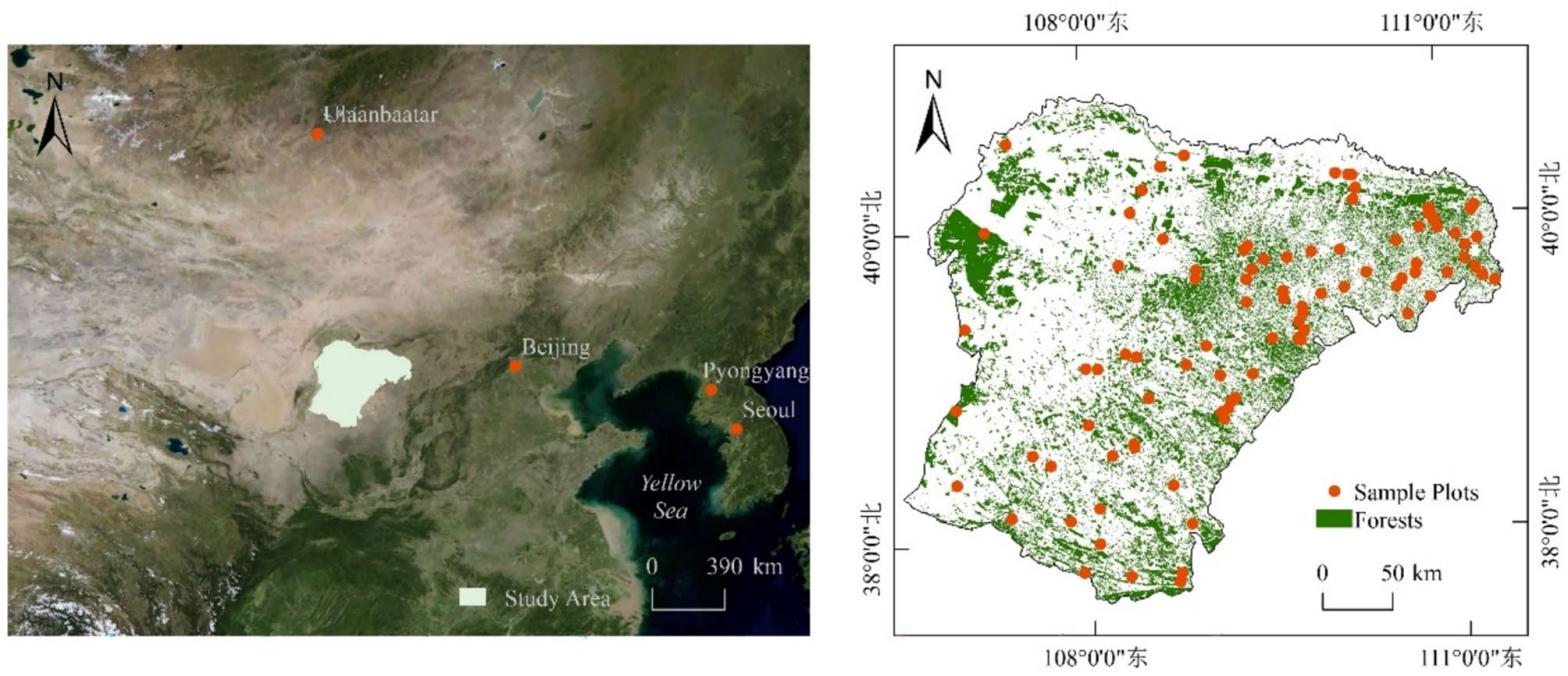
Geographical location of Ordos and spatial distribution of its forests (green areas) and field sampling sites (red dots). (Software: Arcgis 10.8). Data source: Forest vector data for Ordos, originating from the Ordos Forest and Grassland Bureau.

Located in the central part of Inner Mongolia Autonomous Region of China (106° 42′ 40″ E to 111° 27′ 20″ E, 37° 35′ 24″ N to 40° 51′ 40″ N), the Ordos consists of vast desert and grassland areas (Fig. 1). The region has a dry temperate continental climate (BWk, according to the Koppen and Geige climate classification) with an annual mean temperature ranging between 5.3 and 8.7 °C, and an annual mean precipitation between 190 and 400 mm. The elevation of the terrain varies from 850 to 2150 m. The predominant vegetation includes shrubs adapted to arid conditions and species and types such as *Populus*, *Sali*x, and *Pinus tabuliformis*, especially the drought-resistant *Pinus tabuliformi*s, which is commonly used for windbreaks and soil quality improvement ^21^.

### Data collection

#### Field survey

Owing to the arid climate in the study area, the area fraction covered by shrubs significantly exceeded that covered by trees, making shrubs a crucial component of forest carbon storage. Therefore, in this study, we considered shrubs as part of the Ordos Forest, and the shrub carbon storage mentioned hereafter was considered as part of the forest carbon storage. Based on the data of dominant species, types, and shrubs provided by the Forestry and Grassland Science Research Institute of Ordos for 2018, we classified the forest communities into five types: *Populus*, *Salix*, *Pinus tabuliformis*, other species, and shrubs, and conducted field surveys of these communities from July to September 2023(The main shrub species collected were *Caragana korshinskii*, *Hedysarum scoparium*, *Artemisia ordosica*, and *Salix psammophila*.). For trees, each plot covered an area of 20 × 20 m^2^, and for shrubs, it covered 10 × 10 m^2^. For grasslands, each plot consisted of three 1 × 1 m^2^ quadrats for the simultaneous collection of data on the herbaceous layer. During the surveys, the coordinates of five points (four corners and the center) were recorded using a GPS device. All the trees and shrubs in the plots were numbered. For trees, measurements were taken of the species, diameter at breast height (DBH at 1.3 m), height, and crown width. For shrubs, measurements were taken of species, crown width, and height. For grasslands, aboveground herbaceous parts were collected along with root samples using a root borer to a depth of 30 cm at three different locations within each quadrat. In total, 20 *Populus* plots, 18 *Salix* plots, 20 *Pinus tabuliformis* plots, 18 plots of other species and types, and 20 shrub plots were established (Fig. 1). Table 1 shows the carbon density distributions across different types of sample plots (Table 1). To post process the plot data, the biomass of individual trees and shrubs was first computed based on the established models. The carbon storage for each tree was then calculated using carbon coefficients specific to different species and types. For the herbaceous component, the aboveground portions of the collected herbaceous plants were brought back to the laboratory and dried in a 65 °C oven for 48 h. The belowground roots of the herbaceous plants were washed, dried, and weighed to obtain the dry biomass weight. Subsequently, the herbaceous carbon storage was determined using herbaceous-specific carbon coefficients. Finally, the carbon storage of all trees and herbaceous plants within each plot were summed to compute the forest carbon storage for each plot.

**Table 1:** Carbon density estimated for the 96 surveyed plots (unit: t/ha).

Classification scale		Number of plots	Mean value (t/ha)	Minimum value (t/ha)	Maximum value (t/ha)
Dominant species and types species and types	*Populus*	20	32.31	4.04	70.70
	*Salix*	18	24.01	2.09	65.87
	*Pinus tabuliformis*	20	18.25	8.73	73.86
	Other species and types	18	23.31	6.53	45.03
	Shrub	20	9.13	3.36	15.69
Whole forest	–	96	20.62	2.09	70.70

#### Remote sensing data and preprocessing

In this study, we used remote-sensing data from Sentinel 2A and Landsat 8, which have different resolutions.

Sentinel 2, developed by the European Space Agency (ESA), is part of the Copernicus program. The Sentinel 2 mission comprises two similar satellites: Sentinel 2A and Sentinel 2B. Sentinel 2A, launched on June 23, 2015, is equipped with a multispectral instrument capable of capturing 13 different spectral bands, ranging from visible light to near-infrared and short-wave infrared. Sentinel 2 B, launched on March 7, 2017, aims to work in conjunction with Sentinel 2A to enhance data coverage and revisit frequency. The technical specifications of Sentinel 2 B are identical to those of Sentinel 2A. Sentinel 2A has a swath width of 290 km and a revisit time of 10 days. It includes four bands at 10 m resolution covering the visible and near-infrared (NIR) spectra, six bands at 20 m resolution covering the red edge to short-wave infrared (SWIR) range, and three bands at 60 m resolution dedicated to atmospheric correction. In this study, the Sentinel 2A level 2A (L2A) product was selected. Sentinel 2A Level 1C data from July to October 2023 were acquired and preprocessed to obtain images with a cloud cover of less than 5% and ensure that there were no large areas of shadows. Atmospheric correction was performed using the Sen2cor plugin released by the ESA, followed by resampling of all bands to 10 m using SNAP, an image processing package developed by the ESA for handling sentinel data. Finally, the vegetation indices required for this study were calculated.

Landsat 8 is operated by the United States Geological Survey (USGS) and the National Aeronautics and Space Administration (NASA) and is a component of the Landsat program. Launched on February 11, 2013, Landsat 8 was equipped with two primary instruments: an Operational Land Imager (OLI) and a Thermal Infrared Sensor (TIRS). The Landsat 8 OLI had a swath width of 185 km and a revisit time of 16 days. The imagery encompasses 11 spectral bands with a spatial resolution of 30 m. In this study, Landsat 8 OLI data were obtained from May to October 2013 and from June to September 2023. The cloud cover was ensured to be less than 5%, and there were no large shadow areas. The original remote sensing images were pre-processed using ENVI 5.3 software. The preprocessing steps included terrain, geometric, radiometric, and atmospheric corrections. Finally, the vegetation indices used in this study were calculated.

#### Calculation of carbon storage at plot scale

We conducted a comprehensive assessment of the carbon storage capacity of forest ecosystems, which covers both the aboveground and belowground carbon storage of trees and shrubs, and the carbon storage of herbaceous layer plants and their root systems. Thus, the calculation of forest carbon storage in this study included an estimation of carbon storage for each tree, shrub, and herbaceous plant within each plot. For individual trees, the diameter at breast height (DBH) and tree height (H) are critical parameters, and research has shown that the quantitative relationship between DBH and height can effectively predict forest biomass, which can then be used to calculate carbon storage ^22,23^. Similarly, biomass estimation for shrubs generally relies on crown width (C) and height (H). The biomass of the herbaceous layer was obtained by collecting samples from the above-and belowground parts and drying them. When applied to calculate the carbon storage of individual trees, shrubs, and herbaceous plants. Initially, individual carbon storage estimation was performed for all trees, shrubs, and herbaceous plants within each plot, and the data were aggregated to derive the total carbon storage for the plot.

The biomass relationship equations and root-to-shoot ratios for the main species and types, shrub species and types, and herbaceous species are shown in Table 2. The corresponding carbon coefficients, also listed in Table 2, are calculated based on China’s "Guidelines for Carbon Sequestration Measurement and Monitoring in Afforestation Projects (LY/T 2988-2018)”. For tree and shrub species for which the carbon content has not been directly measured, a reference method was used, substituting the carbon content rates with those of similar tree and shrub species.

**Table 2:** Allometric growth equations and carbon coefficients for different tree and shrub species.

Plant species	Nutritional organs	Accumulation-biomass equation	Carbon content
*Populus*	Trunk	$W=0.0859\times {({D}^{2}H)}^{0.8001}$	0.48
	Branch	$W=0.0036\times {({D}^{2}H)}^{0.8437}$	
	Leaf	$W=0.0291\times {({D}^{2}H)}^{0.6136}$	
	Root	$W=0.0143\times {({D}^{2}H)}^{0.8772}$	
*Salix*	Trunk	$W=0.0535\times {({D}^{2}H)}^{0.8977}$	0.47
	Branch	$W=0.0064\times {({D}^{2}H)}^{0.9495}$	
	Leaf	$W=0.0070\times {({D}^{2}H)}^{0.7347}$	
	Root	$W=0.0253\times {({D}^{2}H)}^{0.8435}$	
*Pinus tabulaeformis*	Trunk	$W=0.0455\times {({D}^{2}H)}^{0.8716}$	0.50
	Branch	$W=1.1125\times {({D}^{2}H)}^{0.3455}$	
	Leaf	$W=0.0280\times {({D}^{2}H)}^{0.6399}$	
	Root	$W=0.0191\times {({D}^{2}H)}^{0.8682}$	
*Ulmus pumila*	Trunk	$W=0.0455\times {({D}^{2}H)}^{0.8716}$	0.48
	Branch	$W=0.0814\times {({D}^{2}H)}^{0.7510}$	
	Leaf	$W=0.0178\times {({D}^{2}H)}^{0.7584}$	
	Root	$W=0.0772\times {({D}^{2}H)}^{0.8176}$	
*Pinus sylvestris var. mongholica*	Trunk	$W=0.0805\times {({D}^{2}H)}^{0.8063}$	0.41
	Branch	$W=0.0669\times {({D}^{2}H)}^{0.6268}$	
	Leaf	$W=0.0961\times {({D}^{2}H)}^{0.6553}$	
	Root	$W=0.2385\times {({D}^{2}H)}^{0.5227}$	
*Caragana korshinskii*	Whole	$W=0.90\times {V}^{1060.59}$	0.47
*Artemisia ordosica*	Whole	$W=0.061+2.854e-6\left(HM\right){51.622(HM)}^{2}$	0.47
*Salix cheilophila*	Whole	$W=0.90\times {V}^{1060.59}$	0.47

*W*, biomass; *D*, diameter at breast height;* H*, height; *M*, crown width; *V*, crown volume.

#### 2013 land use classification of ordos

**Fig. 2:**
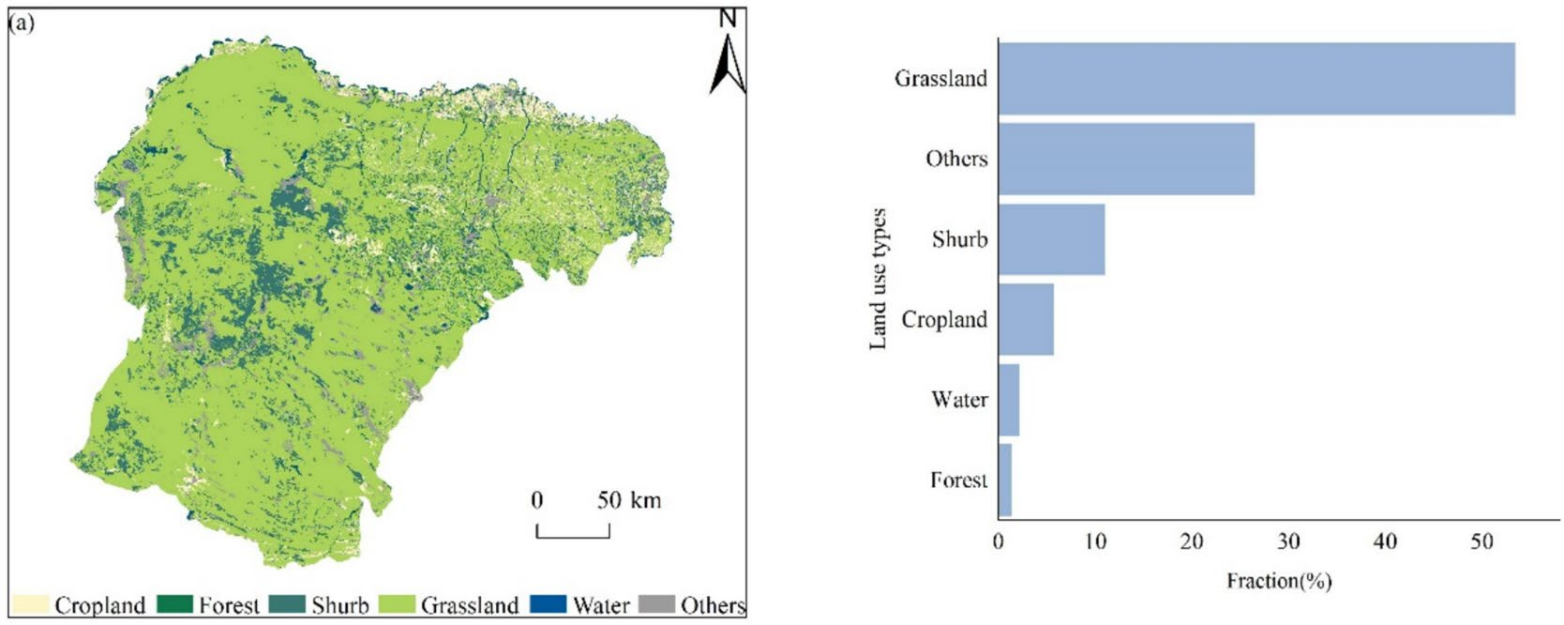
Land use classification and the proportion of each land use type, in Ordos, 2013 (Software: Arcgis 10.8).

Landsat 8 imagery was classified using the maximum likelihood classification method to categorize the types of land use in Ordos in 2013. This is a statistical approach that relies on prior knowledge of the different land cover categories. For this study, land-use types in Ordos in 2013 were categorized into six major classes: cropland, forest, shrub, grassland, and water. The land use classification of Ordos in 2013 using the maximum likelihood method achieved an overall accuracy of 76.8% and a kappa coefficient of 0.71. With this classification, the resulting images clearly displayed the spatial distribution and fraction of land use (Fig. 2), facilitating the extraction of the distribution of forests and shrubs, and the estimation of forest carbon storage.

## Methodology

### Methods for estimating forest carbon storage

#### Remote sensing variable selection

Careful selection of remote-sensing vegetation indices is crucial for carbon storage modeling. We extracted 16 candidate vegetation indices from Sentinel 2A data and 12 from Landsat 8 data. The Pearson correlation coefficients between the remote sensing vegetation indices and measured carbon storage were calculated. These coefficients were ranked to select highly correlated vegetation indices as predictors for carbon storage modeling.

To enhance model stability and generalization ability, this study optimized and screened remote sensing vegetation indices. Pearson’s correlation analysis was used to assess the correlation between the vegetation indices, spectral features, and measured forest carbon density. Variables with a coefficient of determination greater than 0.5 were selected as final modeling factors. The formula used to calculate the Pearson correlation coefficient is as follows:

$$r=\frac{{\sum }_{i=1}^{n}({x}_{i}-\overline{x })({y}_{i}-\overline{y })}{\sqrt{{\sum }_{i=1}^{n}{({x}_{i}-\overline{x })}^{2}}\sqrt{{\sum }_{i=1}^{n}{({y}_{i}-\overline{y })}^{2}}}$$

where *r* represents the correlation coefficient between variables *x* and *y*, *xi* and *yi* represent the sample values in each group of variables, $\bar{x}$ and $\bar{y}$ represent the mean values of each group of variables, *n* represents the sample size. A positive or negative value of *r* indicates the positive or negative correlation between the two groups of variables, respectively. The range of *r* is between − 1 and 1. The closer |*r*| is to 1, the stronger the correlation between the two groups of variables.

Among the modeling factors that are highly correlated with carbon density, particularly among the various remote sensing feature variables, a high correlation may occur. This may have led to multicollinearity problems in the multiple linear regression analysis. Therefore, this study used SPSS to calculate the variance inflation factor (VIF) between the remote sensing feature variables to detect multicollinearity. When the VIF of a modeling factor exceeded 10, collinearity occurred, and we removed the variable.

$$VIF=\frac{1}{1-{R}_{j}^{2}}$$

where ${R}_{j}$ is the negative correlation coefficient obtained by performing regression analysis between the *j*th independent variable and the dependent variable using other independent variables.

Finally, we selected two types of remote sensing imagery (Landsat 8 and Sentinel 2A) and seven groups of remote sensing vegetation indices under two different forest classification scales (dominant species and types and the whole forest) as remote sensing variables for forest carbon storage modeling (Table 3).

**Table 3:** Remote sensing vegetation indices selected for modeling forest carbon storage.

Sensor	Classification scale		Remote sensing variable	Formulae	Pearson correlation coefficient
Sentinel 2A	Dominant species and types	*Populus*	SAVI	$$\begin{gathered} \left( {b8 - b4} \right) \times (1 + L)/b8 \hfill \\ + b4 + L \hfill \\ \end{gathered}$$	0.734
			NDVI	$(b8-b4)/(b8+b4)$	0.734
			gNDVI	$(b8-b3)/(b8+b3)$	0.723
		*Salix*	DVI	$b8-b4$	0.632
			gNDVI	$(b8-b3)/(b8+b3)$	0.630
		*Pinus tabulaeformis*	reNDVI	$(b8A-b5)/(b8A+b5)$	0.654
			SR	$b8/b4$	0.674
			ARVI	$$\begin{gathered} b8 - (2 \times b4 - b2)/b8 \hfill \\ + (2 \times b4 - b2) \hfill \\ \end{gathered}$$	0.674
		Other species and types	NDVI	$(b8-b4)/(b8+b4)$	0.554
			SAVI	$$\begin{gathered} \left( {b8 - b4} \right) \times (1 + L)/b8 \hfill \\ + b4 + L \hfill \\ \end{gathered}$$	0.554
			ARVI	$$\begin{gathered} b8 - (2 \times b4 - b2)/b8 \hfill \\ + (2 \times b4 - b2) \hfill \\ \end{gathered}$$	0.572
		Shrub	RDVI	$b8-b4/\sqrt{b8+b4}$	0.599
			SR	$b8/b4$	0.612
			reSR	$b8A/b5$	
Landsat 8 (approach 1)	Whole forest	Whole forest	gNDVI	$(b5-b3)/(b5+b3)$	0.487
			NDVI	$(b5-b4)/(b5+b4)$	0.358
Landsat 8 (approach 2)	Whole forest	Whole forest	NDVI	$(b5-b4)/(b5+b4)$	0.458
			SAVI	$$\begin{gathered} \left( {b8 - b4} \right) \times (1 + L)/b8 \hfill \\ + b4 + L \hfill \\ \end{gathered}$$	0.498
			gNDVI	$(b5-b3)/(b5+b3)$	0.537

In Sentinel 2A imagery: b2 is the Blue band, b3 is the Green band, b4 is the Red band, b5 is Red Edge band 1, b8 is the NIR band, and b8A is the Narrow NIR band. In Landsat 8 imagery: b3 is the Green band, b4 is the Red band, b5 is the NIR band, and b8 is the Panchromatic band.

#### Methods for carbon storage modeling

In this study, Random Forest (RF), Decision Tree (DT), and Multiple Linear Regression (MLR) were used to estimate carbon density per pixel across different spatial resolutions (Sentinel 2A and Landsat 8) and forest classification scales (dominant species and types and whole forest). A random sampling approach was used to split the dataset into training and test sets in a 7:3 ratio for each model. Subsequently, each model was trained, and its performance was evaluated by calculating the R^2^ score, root mean square error (RMSE), relative root mean square error (rRMSE), and mean absolute error (MAE). The performances of the different models were compared for different remote sensing data resolutions and forest classification scales.

Random Forest (RF) is a machine-learning algorithm built on the principles of ensemble learning^16^. This algorithm integrates multiple independent regression trees into a "forest,” where each tree is trained on a randomly drawn subset of the data and models the relationships between input variables and target carbon storage values It can capture complex non-linear relationships in the data, particularly excelling in ecosystem models with many vegetation indices and intricate data structures, thus offering higher predictive accuracy and robustness than simple linear regression. In multivariate analyses, the RF diversity of decision trees and ensemble strategy effectively prevented overfitting, ensuring that the model maintained good generalization across various datasets.

For this study, we applied a random forest model, using multiple remote sensing feature variables from Landsat 8 and Sentinel 2A remote sensing data and measured forest carbon density as training data, to establish carbon density estimation models under two types of remote sensing imagery and two classification scales, the random forest model training parameters were set as follows: n_estimators = 100, max_depth = 5, min_samples_split = 3, random_state = 42.

Decision trees (DT) are supervised learning algorithms widely used in classification and regression tasks^24^. They operate by analyzing features and recursively partitioning the dataset into more homogeneous subsets, forming a tree structure that simulates the decision-making process. During this process, the internal nodes test the feature values, branches represent the outcomes, and the leaf nodes deliver the predictive results. The algorithm assesses the splitting effectiveness of each feature by selecting points that maximize information gain, minimize Gini impurities, or reduce variance. This process continues until the predetermined stopping criteria are met, gradually constructing a decision tree that reflects the structure of the data and enhances prediction accuracy.

Herein, we applied a decision tree model, using multiple remote sensing feature variables from Landsat 8 and Sentinel 2A remote sensing data and measured forest carbon density as training data, to establish carbon density estimation models under two types of remote sensing imagery and two classification scales, the decision tree model training parameters were set as follows: max_depth = 4, min_samples_split = 3, random_state = 42.

The MLR model aims to find a linear equation that best describes the relationship between independent and dependent vegetation indices. Its simplicity and interpretability render it suitable for datasets with clearly inherited linear relationships^25^. The MLR model can be expressed as:

1 $$\begin{array}{c}y={\beta }_{0}+{\beta }_{1}\times {x}_{1}+{\beta }_{2}\times {x}_{2}+\cdots +{\beta }_{n}\times {x}_{n}+\epsilon \end{array}$$

where *y* is the dependent variable, ${x}_{i}$ is the independent vegetation index, ${\beta }_{i}$ is a fitting parameter, and; *ε* is the error term.

### Carbon storage estimates using Landsat 8 imagery

To estimate the entire forest carbon storage using Landsat 8 imagery, we used two approaches: Approaches 1 and 2. In Approach 1, carbon storage was modeled by directly correlating the measured carbon values with selected Landsat 8 vegetation indices. In Approach 2, carbon storage estimates were derived from high-resolution imagery of dominant species and types, which then served as reference values for modeling using Landsat 8 vegetation indices. In Approach 2, carbon storage estimates obtained from Sentinel 2A imagery (referred to as Result S2) were resampled to a 30 m resolution and integrated into the coarser-resolution Landsat 8 imagery. A Pearson correlation analysis between the results in S2 and the Landsat 8 vegetation indices was then conducted to select the most suitable Landsat 8 vegetation indices for carbon storage modeling.

### Accuracy evaluation

Five metrics were employed to evaluate the model performance, namely, R^2^, RMSE, rRMSE, MAE, and the Mean Bias Error (MBE), defined as

2 $$\begin{array}{c}{R}^{2}=1-\frac{{\sum }_{i=1}^{n}{\left({y}_{i}-{\widehat{y}}_{i}\right)}^{2}}{{\sum }_{i=1}^{n}{{(y}_{i}-\overline{y })}^{2}}\end{array}$$

3 $$\begin{array}{c}RMSE=\sqrt{\frac{{\sum }_{i=1}^{n}{\left({y}_{i}-{\widehat{y}}_{i}\right)}^{2}}{n}}\end{array}$$

4 $$\begin{array}{c}rRMSE=\sqrt{\frac{\frac{1}{n}{\sum }_{i=1}^{n}{\left({y}_{i}-\widehat{y}\right)}^{2}}{\overline{y}}}\end{array}$$

5 $$\begin{array}{c}MAE=\frac{1}{n}\sum_{i=1}^{n}\left|{y}_{i}-{\widehat{y}}_{i}\right|\end{array}$$

6 $$\begin{array}{c}MBE=\frac{1}{n}{\sum }_{i-1}^{n}\left({y}_{i}-{\widehat{y}}_{i}\right)\end{array}$$

where *y*_*i*_ represents the measured and ${\widehat{y}}_{i}$ estimated carbon storage, $\overline{y }$ is the ensemble mean of *y*_*i*_, and *n* is the number of samples^26,27^.

### Geodetector

Geodetection is a statistical technique used to analyze the spatial correlations and interactions between environmental factors and the research target (here, carbon storage). This method is based on the principle of geographic heterogeneity and utilizes statistical differences at the spatial level to identify and explain the influences of environmental vegetation indices on the target. This study aimed to ascertain the explanatory power of environmental factors on the target by comparing the differences among vegetation indices within different geographic subregions. It features four key functions—factor detector, ecological detector, risk detector, and interaction detector—to evaluate the explanatory power of a single factor, analyze the interactions between two factors, identify high-risk areas, and explore the synergistic effects among multiple factors.

## Results

### Models of carbon storage estimation at different spatial resolutions

By comparing the Pearson correlation coefficients, the optimal remote-sensing vegetation indices were selected for modeling forest carbon storage in Ordos (Table3). Subsequently, RF, DT, and MLR models were developed to estimate carbon storage. The evaluation metrics of the carbon storage estimation models for different forest classification scales and remote sensing resolutions for 21 combinations were compared, as shown in Table 4.

**Table 4:** Comparison of carbon storage modeling accuracy across different classification scales and remote sensing data.

Image	Classification scale		Model	R^2^	RMSE	rRMSE	MAE
Sentinel 2A	Dominant species and types	*Populus*	RF	0.66	10.73	0.31	8.29
			DT	0.61	11.48	0.33	9.73
			MLR	0.40	14.20	0.40	12.04
		*Salix*	Random Forest	0.42	14.50	0.54	10.68
			Decision Tree	0.63	11.48	0.43	10.53
			Multiple Linear	0.35	15.31	0.57	10.84
		*Pinus tabulaeformis*	Random Forest	0.48	5.26	0.35	4.04
			Decision Tree	0.53	4.98	0.33	4.17
			Multiple Linear	0.42	5.52	0.36	4.58
		Other Species and types	Random Forest	0.43	6.13	0.29	5.11
			Decision Tree	0.32	7.92	0.37	6.11
			Multiple Linear	0.28	8.94	0.42	7.96
		Shrub	Random Forest	0.70	1.51	0.19	1.34
			Decision Tree	0.25	2.40	0.30	1.87
			Multiple Linear	0.78	1.28	0.16	1.21
Landsat 8 (approach 1)	Whole forest		Random Forest	0.31	12.56	0.56	9.17
			Decision Tree	0.35	12.25	0.54	9.18
			Multiple Linear	0.33	12.48	0.50	8.61
Landsat 8 approach 2)	Whole forest		Random Forest	0.33	6.71	0.45	3.70
			Decision Tree	0.33	6.72	0.53	3.70
			Multiple Linear	0.31	6.81	0.50	3.85

Carbon storage models using Sentinel 2A data for dominant species and types showed higher accuracy. For Sentinel 2A imagery, the RF model provided the highest accuracy for carbon storage estimates of *Populus* trees and other species and types, although the RMSE, rRMSE, and MAE values were lower than those of the DT and MLR models. For *Salix tabulaeformis*, the DT model exhibited higher R^2^ values and lower RMSE, rRMSE, and MAE values than those of the RF and MLR models. The MLR model showed a high R^2^ value for shrubs. The models showed lower accuracy for the Landsat 8 imagery. The R^2^ values for the models constructed using Approaches 1 and 2 were not significantly different. However, the RMSE, rRMSE, and MAE values for approach 2 were consistently lower than those for approach 1. For example, the RMSE and MAE of approach1 were approximately one-third of those of approach 2.

**Fig. 3:**
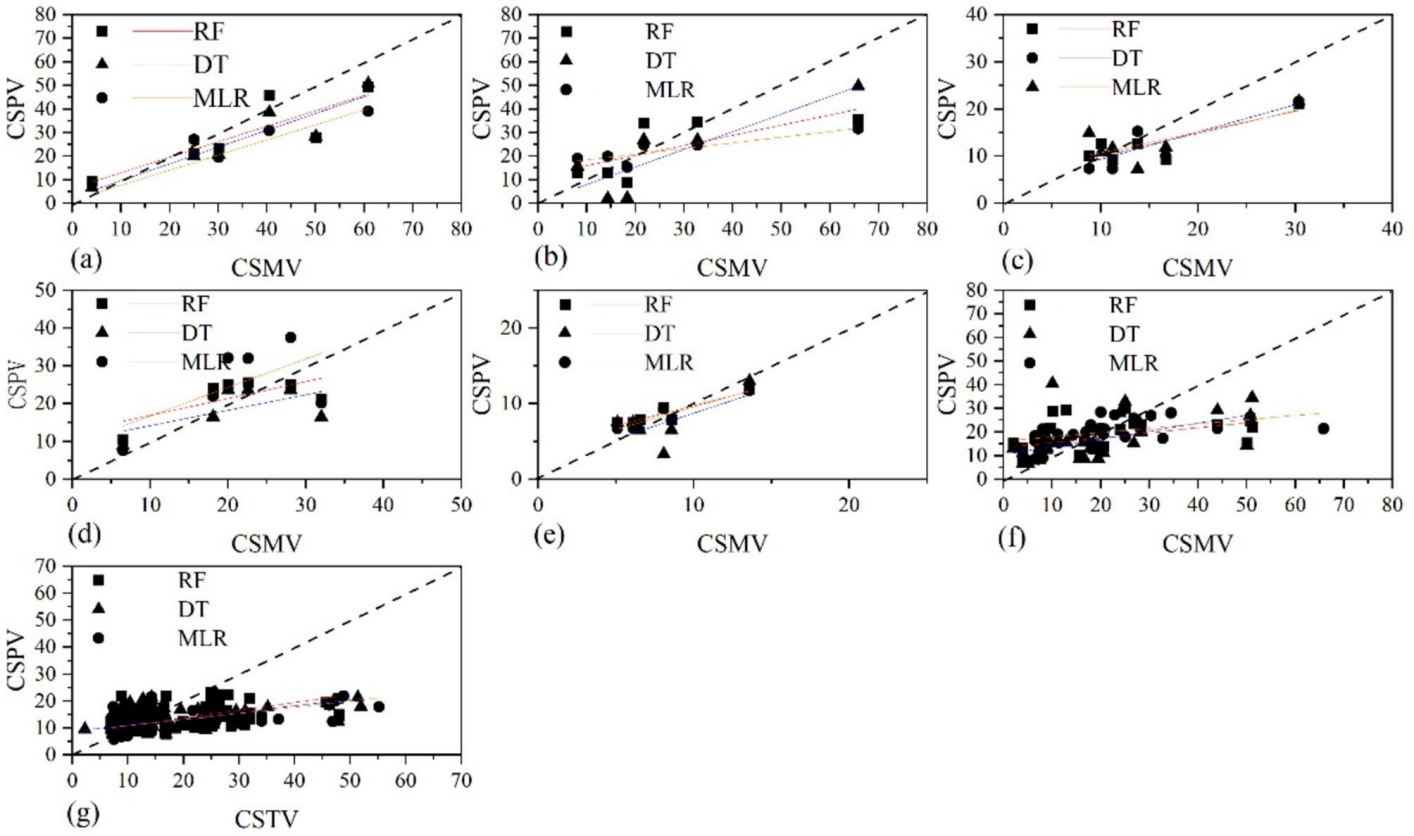
Scatter plot between measured and predicted forest carbon density (the black dotted line indicates y = x). (t/ha). (**a**) Scatter plot of observed and predicted carbon densities for *Populus* trees using three models based on Sentinel-2 imagery. (**b**) as (**a**), but for *salix* trees; (**c**) as (**a**), but for pinus tabulaeformis; (**d**) as (**a**), but for other species and types; (**e**) as (**a**), but for shrub; (**f**) Scatter plot of observed and estimated carbon density for the entire forest using three models based on Landsat 8 imagery under approach 1. (**g**) Scatter plot of “true” and estimated carbon density for whole forest using three models and based on Landsat 8 imagery under the approach 2 (Note: CSMV is the measured carbon storage value, CSPV is the predicted carbon storage value, and CSTV is the carbon storage reference value based on Result S2).

Scatter plots (Fig. 3) show the observed and predicted carbon storage from the test set during the model development process.

### Spatial differences in carbon storage estimation with two approaches based on Landsat 8 imagery.

**Fig. 4:**
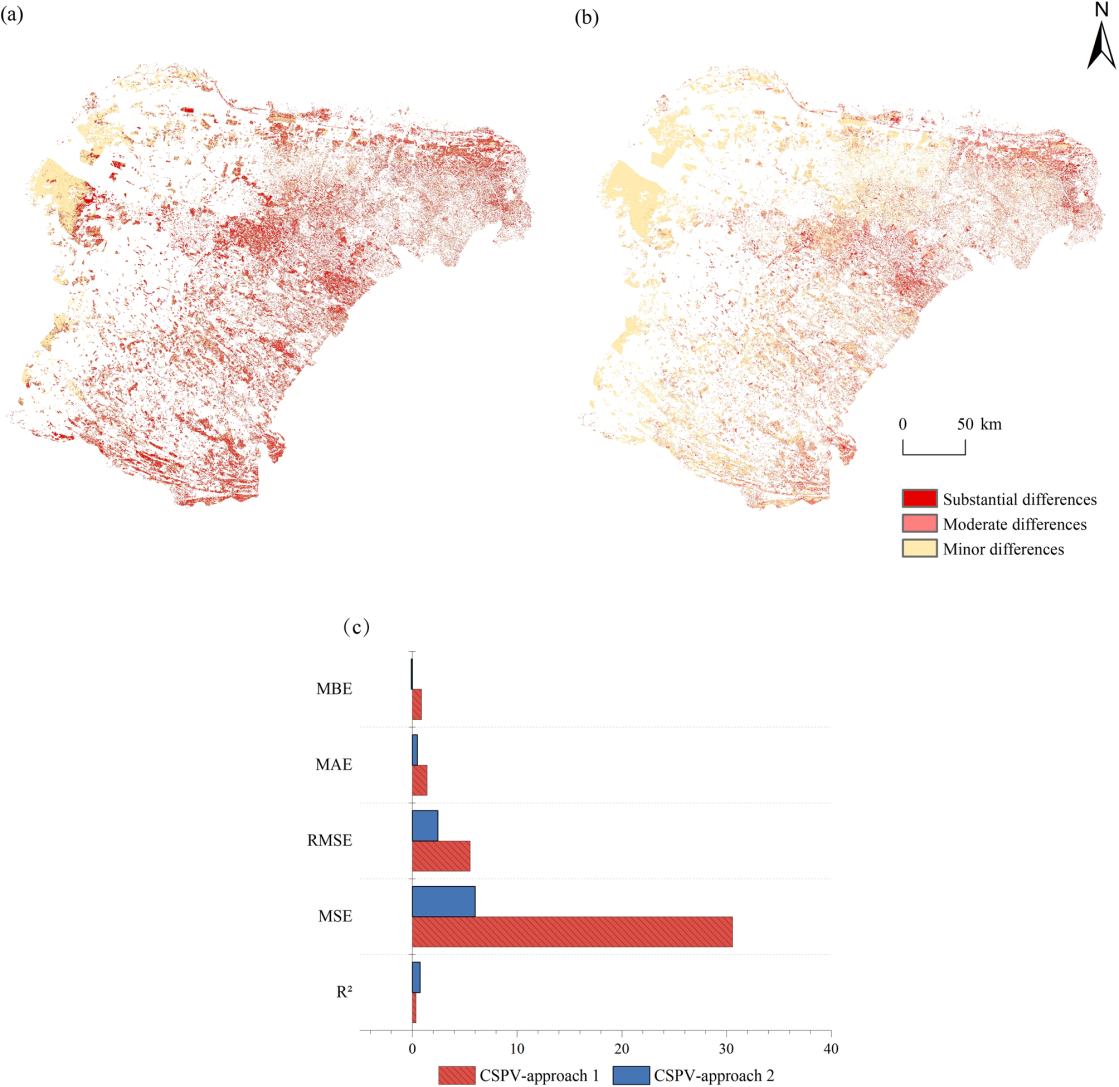
Spatial distribution and accuracy comparison of differences under the approach 1 and approach2 (Software: Arcgis 10.8). (**a**) Spatial distribution of differences in carbon storage estimates between approach 1 and Sentinel 2A imagery. (**b**) as (**a**), but for approach 2; (**c**) Accuracy comparison of carbon storage estimates under approaches 1 and 2 compared to result S2 (CSPV-approach 1 represents the predicted carbon storage values based on Landsat 8 imagery under approach 1; CSPV-approach 2 represents the predicted carbon storage values based on Landsat 8 imagery under approach 2).

Using Landsat 8 imagery, the forest carbon storage was estimated for the entire forest, and the results of approaches 1 and 2 were compared. We defined three levels of discrepancies in carbon storage estimates: (1) “substantial difference,” a discrepancy in carbon storage exceeding 60% between the estimates using the Landsat 8 and the Sentinel 2A; (2) “moderate difference,” a discrepancy in carbon storage between 30 and 60%; and (3) “minor difference,” a discrepancy in carbon storage less than 30%. For both Approach 1 and Approach 2, regions of “minor differences” in forest carbon storage exceeded 90%. For Approach 2, the area of “substantial differences” decreased by 4% compared to approach1, and the area of “minor differences” reached 95%, 3% higher than for approach 1 (Table 5). Figure 4a,b show that the area of “moderate differences” for approach 2 largely coincides with the “substantial differences” area under Approach 1. Figure 4c shows a comparison of the accuracy between the whole-forest carbon storage estimates from Approach 1 and Approach 2, with result S1. R^2^, MSE, RMSE, MAE, and MBE of Approach 1 were superior to those of Approach 2. This indicates that Approach 2 significantly reduces estimation biases.

**Table 5:** Percentage differences in carbon storage estimates using two approaches with Landsat 8 compared to result S2.

Method	Substantial differences (%)	Moderate differences (%)	Minor differences (%)
Approach 1	5.69	1.98	92.32
Approach 2	1.37	3.51	95.12

### Spatial distribution of forest carbon density and carbon storage

#### Spatial distribution of carbon storage of dominant species and types based on Sentinel 2A imagery

**Fig. 5:**
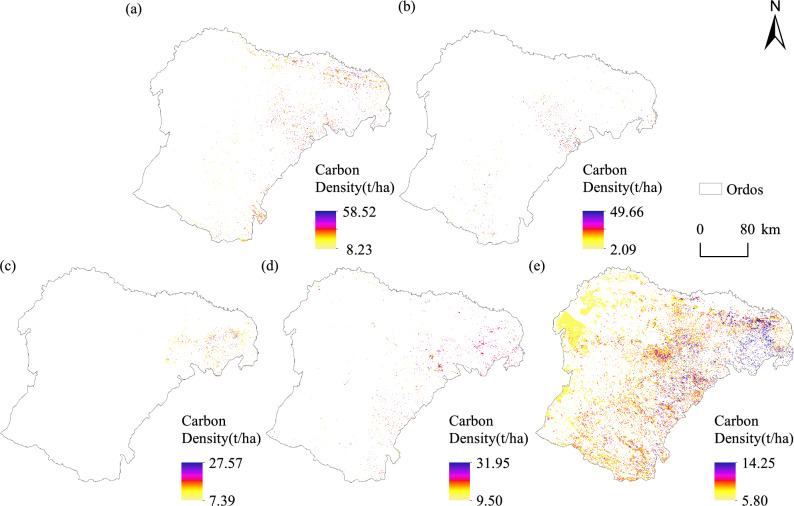
Spatial distribution of forest carbon density for different dominant species and types (Software: Arcgis 10.8) (**a**) Spatial distribution of forest carbon density for *populus*; (**b**) as (**a**), but for *salix*; (**c**), as in (**a**), but for *P. tabuliformis*. (**d**), as in (**a**), but for other species and types. (**e**) as (**a**), but for shrub.

The spatial distribution of forest carbon density for the dominant species and types in Ordos is shown (Fig. 5 and Table 6). *Populus* trees had an average carbon density of 29.14 t/ha, with values ranging from 8.23 to 58.82 t/ha. The distribution was relatively uniform, and the total carbon storage was 4.5 Mt, accounting for 17.38% of the total carbon storage, which was the largest among all the tree types. *Salix* trees had an average carbon density of 27.39 t/ha, with values ranging from 2.09 49.66 t/ha. The total carbon storage for willows was 1,197,381.528 t, accounting for 4.62% of the total carbon storage. *Pinus tabuliformis* distributed primarily in the Jungar Banner of Ordos, has an average carbon density of 13.00 t/ha, with values ranging from 7.39 to 27.57 t/ha. With a total carbon storage of 825,591.2087 t, accounting for 3.18% of the total forest carbon storage. The other species and types have an average carbon density of 23.24 t/ha, with values ranging from 9.50 to 31.95 t/ha. Their total carbon storage is 2.15 Mt, accounting for 8.30% of the total forest carbon storage. Shrubs have an average carbon density of 9.02 t/ha, with values ranging from 5.80 to 14.25 t/ha. The total carbon storage for shrubs is 17.25 Mt. Despite their low carbon density, shrubs are the main contributor to the total carbon storage, accounting for 66.52% due to their large fraction of coverage.

**Table 6:** Carbon density and total carbon storage for different dominant species and types.

Dominant species and types	Carbon density (t/ha)			Total carbon storage(t)	Percentage (%)
	Mean	Min	Max		
*Populus*	29.14	8.23	58.52	4,506,024.85	17.38
*Salix*	27.39	2.09	49.66	1,197,381.53	4.62
*Pinus tabuliformis*	13.00	7.39	27.57	825,591.21	3.18
Other Species and types	23.24	9.50	31.95	2,150,570.97	8.30
Shrub	9.02	5.80	14.25	17,245,919.68	66.52

#### Spatial distribution of forest carbon density in different years

**Fig. 6:**
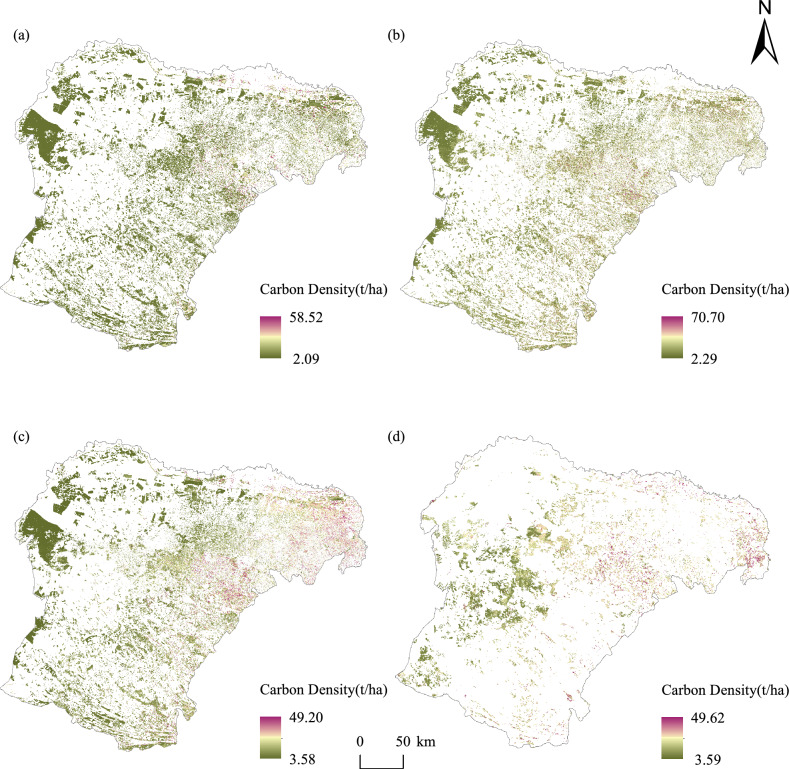
Spatial distribution of whole forest carbon density at different spatial resolutions for different years (Software: Arcgis 10.8). (**a**) the spatial distribution of whole forest carbon density based on sentinel 2A imagery in 2023; (**b**) the spatial distribution of whole forest carbon density based on approach 1 using Landsat 8 imagery in 2023; (**c**) as (**b**), but for approach 2; (**d**) the spatial distribution of whole forest carbon density based on approach 2 using Landsat 8 imagery in 2013.

For 2023, using Sentinel 2A imagery, we aggregated the carbon storage of all dominant species and types in Ordos to obtain the precise spatial distribution of carbon storage in the entire forest (Fig. 6a). Using Landsat 8 imagery, we applied Approaches 1 and 2 to estimate the carbon storage of the entire forest at lower resolution (Fig. 6b,c). In 2013, we extracted forest and shrub areas from the land use classification of Ordos, and using the random forest model under Approach 2 combined with NDVI, SAVI, and gNDVI from Landsat 8 imagery, we modeled the carbon storage for Ordos, resulting in a precise spatial distribution of forest carbon storage for 2013 (Fig. 6d).

For 2023, using Sentinel 2A imagery, the whole forest carbon storage in Ordos was about 26 Mt, with an average carbon density of 20.36 t/ha, and a range from 2.09 to 58.52 t/ha (Table 7). Carbon density distribution was relatively uniform across the region, with shrubs being the predominant contributors. Using the Approach 1, the whole forest carbon storage estimated about 40.54 Mt, with an average carbon density of 20.56 t/ha, ranging from 2.29 to 70.70 t/ha. Using the Approach 2, the carbon storage for 2013 was about 26.74 Mt, with an average carbon density of 19.78 t/ha, ranging from 3.59 to 49.20 t/ha (Table 7).

**Table 7:** Values of whole forest carbon density and carbon storage at different spatial resolutions for different years.

Year	Image	Carbon density (t/ha)			Total carbon storage (t)
		Mean	Min	Max	
2023	Sentinel 2A	20.36	2.09	58.52	25,925,488.23
	Landst 8 (approach 1)	20.56	2.29	70.70	40,544,080.50
	Landst 8 (approach 2)	19.78	3.59	49.2	26,735,429.73
2013	Landst 8 (approach 2)	16.55	2.59	49.62	13,715,729.12

Approach 2 significantly outperformed Approach 1 in terms of accuracy in estimating the total carbon storage. The difference between the total carbon storage estimated using Landsat 8 with Approach 2 and that using Sentinel 2A was approximately 3%. This difference increased to 36% for approach 1. The carbon density range for Approach 1 exceeded that estimated using Sentinel 2A and the dominant species and types, with a maximum of 70.70 t/ha. The carbon density range for approach 2 was lower than that estimated using Sentinel 2A, with a maximum of 49.2 t/ha. Overall, Approach 1 exhibited a tendency toward overestimation, primarily because of three factors: the limited spatial representativeness of ground samples with extensive carbon density ranges, mixed pixel effects in vegetation-dense regions at lower resolution, and potential modeling bias from spatially heterogeneous sampling distribution. Conversely, Approach 2 demonstrated a systematic underestimation attributable to the utilization of Sentinel 2A estimates as reference values with enhanced vegetation detail detection, information loss during resolution resampling, and the inherently conservative nature of predictions based on high-resolution reference data. However, Approach 2 demonstrated superior performance as its estimates aligned more closely with the high-resolution Sentinel 2A results, suggesting enhanced reliability in carbon storage estimation. The results of whole-forest carbon storage estimated using Approach 2 were superior to those obtained using Approach 1, which is consistent with the findings reported in "Spatial differences in carbon storage estimation with two approaches based on Landsat 8 imagery.".

With Approach 2, the carbon storage for whole forest in 2013 was about 13.72 Mt, with an average carbon density of 16.55 t/ha and a carbon density range of 2.59 to 49.62 t/ha. Over the decade 2013–023, the forest carbon storage of Ordos increased by approximately 12.21 Mt or 89%. However, it is noteworthy that by comparing the forest carbon density distribution maps of Ordos in Fig. 6c (2023) and Fig. 6d (2013), the green low carbon density zones in 2013 significantly diminished or disappeared by 2023. This transformation may be attributed to land use type conversion, whereby these areas have shifted from previously low-carbon density forestland to other land use types, such as agricultural development, urban construction, or vegetation reduction resulting from natural disasters. Overall, the forest carbon storage in Ordos showed a significant increasing trend. This increase can be attributed to the extensive tree-planting and grassland restoration projects implemented over the decade, including the Desertification Prevention and Control Quality Improvement Action, Soil and Water Conservation Ecological Management, Wetland Protection and Restoration, and Natural Reserve Environmental Restoration projects, which improved the environmental quality and enhanced the region’s carbon sequestration capacity.

## Discussion

### Accuracy of machine learning algorithms

Machine learning significantly enhances the speed and efficiency of the processing and interpretation of large-scale scale remote sensing datasets^28^. It has proven superior in tasks, such as image classification using algorithms such as SVM, Random Forest, and Deep Learning to develop more precise predictive models than traditional methods^29–32^. Most machine learning models presented here for carbon storage estimation exhibited higher R^2^ values than multiple linear regression, because machine learning models can better handle high-dimensional and non-linear data, which are characteristic of complex forest ecosystems. The superiority of the machine learning models was also observed in terms of RMSE, rRMSE, and MAE. However, for shrub vegetation, the multiple linear regression models showed better accuracy in estimating carbon storage. This is due to the characteristics of the shrubs in Ordos, which are mostly artificially planted with uniform species and densities. This uniformity simplified the multiple linear regression to effectively capture the relationships between carbon storage and the predictors. Hence, under certain circumstances, the traditional models may be a good choice ^33^. This suggests that when applying machine learning models to a study such as ours, the characteristics of different vegetation types should be considered.

### Advantages and limitations of high-resolution reference-based carbon storage estimation

The use of high-resolution remote sensing imagery and refined forest classification methods has significantly enhanced the accuracy of forest carbon storage estimations^34^. In particular, modeling and estimating forest carbon storage at the scale of dominant species and types often exhibits greater precision compared to the estimates of forest carbon storage made at the general classification scale of the whole forest. Compared the accuracy of carbon storage estimates using dominant species and types in an entire forest. Their research demonstrated that classification methods focusing on dominant species were more effective at improving the accuracy of carbon storage estimations, especially within highly diverse forest ecosystems. Explored the application of high-resolution imagery to identify dominant species and types to improve carbon assessments in forests with high conservation value^35^. Their findings indicate that accurate identification of dominant species significantly enhances the precision and reliability of carbon storage estimations by employing machine learning techniques to classify dominant species and types in forests and analyze the impact of this approach on the precision of carbon storage estimations^36^. Their results confirmed that machine learning-assisted classification of dominant species and types provides more accurate carbon storage estimates than traditional whole-forest classification methods, particularly for structurally complex forests. These findings are consistent with those of our research, which revealed that models based on high-resolution imagery of dominant species and type classification outperformed those based on lower-resolution whole-forest classification (Approach 1).

**Fig. 7:**
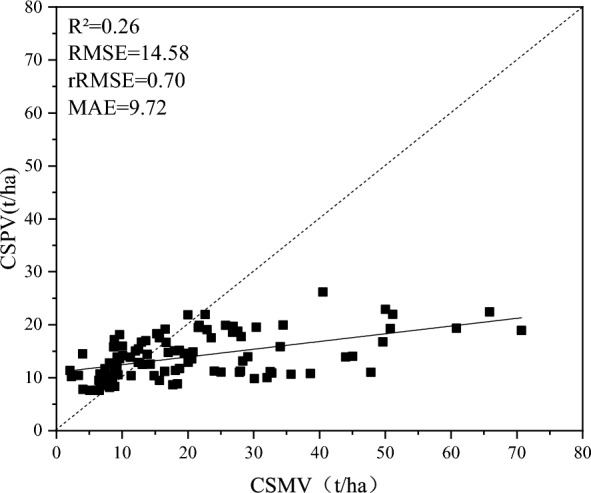
Scatter plot of true values and estimated values of carbon storage using approach 2 compared to observed carbon storage.

Based on the correlations between the Landsat 8 NDVI, SAVI, and gNDVI and the carbon storage reference values (Table 8), we developed a new model for estimating carbon storage (Approach 2). Compared to Approach 1, Approach 2 showed superior performance across all evaluation metrics and closely matched the forest carbon storage estimations derived from S2. However, to validate the consistency of Approach 2 with on-ground measured carbon storage, we found that the fit between Approach 2 estimated carbon storage and actual measurements was low, with an R^2^ of only 0.26, RMSE of 14.58, rRMSE of 0.70, and MAE of 9.72, indicating shortcomings in accuracy. Moreover, when the carbon storage estimated by Approach 2 was approximately 25 t/ha or lower, the fitting curve was closer to y = x. Given that the mean carbon storage from Sentinel 2A based result S2 was approximately 20 t/ha, the carbon storage estimation model using the result of S2 as a reference value performed poorly at higher carbon density values. Therefore, although Approach 2 performed well in predicting the entire forest carbon storage, its precision needs improvement, highlighting the potential limitations of Approach 2 (Fig. 7).

**Table 8:** Correlation between carbon storage estimates from Sentinel 2A and remote sensing variable from Landsat 8.

Feature vegetation indices	gCI	gNDVI	NDVI	NDWI	SAVI	DVI	SR	EVI
Correlation coefficient	0.4093	0.5373	0.4578	− 0.4373	0.4977	0.4226	0.3587	0.4380

### Factors influencing carbon storage in 2013 and 2023

In the context of global climate change and ecological balance, discussing the factors influencing forest carbon storage is crucial for understanding the carbon cycle and formulating effective environmental policies^37^. The determinants of forest carbon storage include natural and socioeconomic factors. These factors not only impact carbon storage individually, but their interplay is also essential for predicting future carbon sequestration potential and developing carbon emission reduction^34,38^. Natural conditions, such as temperature and precipitation, directly influence plant growth, thereby affecting carbon fixation and the carbon storage capacity of forests^39^. Topographical features, such as slope and altitude, indirectly impact forest productivity^40–42^. Socioeconomic activities also significantly affect carbon storage through changes in land use, the promotion of different forms of economic development, and the implementation of policy regulations^43^. For instance, population growth and urbanization typically lead to a reduction in forest areas, whereas different types of economic activities and environmental policies influence the dynamics of regional carbon cycles^44,45^.

**Fig. 8:**
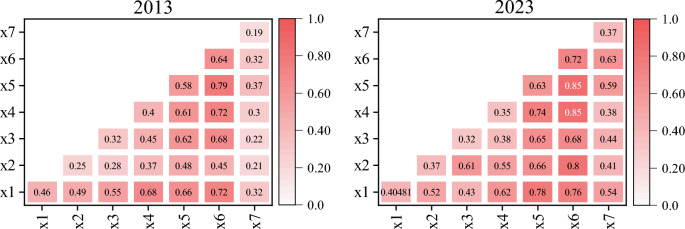
Interactive effects of environmental and socioeconomic factors on forest carbon storage in 2013 and 2023.

To investigate the impact of natural and socioeconomic factors on forest carbon storage in Ordos, we employed Approach 2 to estimate the carbon storage for 2013. Additionally, we used a geodetector to explore how environmental and socioeconomic factors interact over different years to influence carbon storage. Analysis of the interaction matrices from 2013 to 2023 revealed significant impacts of these factors on carbon storage capabilities (Fig. 8). In 2013, aspect (× 6) exhibited the strongest individual impact on carbon storage (0.64), whereas the interactions between temperature (× 4) and DEM (× 1), GDP (× 2), and precipitation (× 3) were 0.55, 0.58, and 0.60, respectively, indicating significant joint effects of these factors on carbon storage. By 2023, the individual impact of aspect on carbon storage increased to 0.72, and its interactions with temperature and slope reached as high as 0.85, reflecting the growing importance of topographic and climatic factors in carbon storage estimation. Additionally, the correlation between GDP and temperature and precipitation strengthened over the decade from 0.58 and 0.68 to 0.62 and 0.74 respectively, suggesting an increasing influence of economic activities on carbon storage under specific climatic conditions. The interaction between population (× 7) and GDP increased from 0.32 in 2013 to 0.37 in 2023, implying a more direct impact of population growth and economic development on carbon storage.

These findings are crucial for the formulation of regional carbon management and climate adaptation strategies. Policymakers must consider the combined effects of economic development, population changes, and geographical factors on carbon storage to implement effective carbon reduction and climate adaptation measures more efficiently. In regions with significant topographic changes, the roles of geographical and climatic factors in carbon storage dynamics should be emphasized to enhance the effective management and growth of carbon reserves. These analyses not only help in understanding the drivers behind changes in carbon storage but also provide data support and a basis for decision-making for future sustainable development. These analyses not only help in understanding the drivers behind changes in carbon storage but also provide data support and a basis for decision-making for future sustainable development.

### Limitations of the research

This study has certain limitations in the carbon storage estimation process for Ordos Forest. First, the sample collection was limited in quantity, particularly for machine learning modeling, where a small sample size may lead to insufficient model training, thereby affecting its generalization ability and prediction stability. Machine learning algorithms typically require large sample datasets to capture complex non-linear relationships, and the limited sample size causes the model to perform poorly when handling outliers and extreme cases, particularly those showing obvious underestimation trends in high carbon density areas. Secondly, our research findings, based on two types of remote sensing images and two classification scales, revealed that the carbon storage estimated using Method 1 based on Landsat 8 imagery at the whole-forest scale (40,544,080.50 t) was significantly higher than that estimated using Sentinel 2A imagery at the dominant tree species scale (25,935,488.24 t), with a difference of approximately 36%. Although the estimation results at the dominant tree species scale based on Sentinel 2A imagery showed better model fitting performance, the inversion values were generally lower than the measured values in the sample plots, with obvious underestimation in high-carbon-density areas. Taking *Pinus tabuliformis* as an example, the measured average carbon density was 18.25 t/ha (range 8.73–73.86 t/ha), while the inversion result average was only 13.00 t/ha (range 7.39–27.57 t/ha); the inversion results for *populus* and *salix* also showed varying degrees of estimation bias. This systematic difference may stem from several aspects: classification scales reflect different levels of forest ecosystem characteristics; remote sensing inversion models tend to average extreme values; scale effects exist between sample plot scale and remote sensing pixel scale; and estimation biases caused by different modeling strategies. These issues suggest that in the estimation of the dominant tree species scale, further improvements in methodological theory research are needed. Future research should focus on the following aspects.

Expanding the sample size and optimizing sampling methods to ensure that samples can adequately represent the carbon storage characteristics of different forest types, with particular attention paid to sampling in high carbon density areas, will improve the machine learning model training effectiveness and prediction accuracy. More complex modeling methods such as ensemble learning, deep learning, and other advanced algorithms can better capture the non-linear relationship between forest carbon density and remote sensing features. Introducing additional auxiliary variables, such as stand structure indices, age distribution, and topographic factors, should be considered to enrich the model inputs and improve the prediction capabilities for areas with high carbon density. Conduct in-depth research on the influence mechanism of classification scale selection on carbon storage estimation and explore the optimal modeling scales. Suitable correction methods should be explored for systematic bias in the estimation of the scale of dominant tree species.

## Conclusions

In this study, we developed forest carbon storage estimation models based on two types of remote sensing imagery and two forest classification scales (dominant species and types and the entire forest), and compared the accuracy of the estimated carbon storage. Subsequently, using the high-accuracy inversion results and dominant species and types as reference values, carbon storage estimates were obtained using Approach 2. The forest extent in 2013 was extracted and Approach 2 was applied to estimate the entire forest carbon storage in Ordos for that year. The important findings are as follows:

Based on optimal remote-sensing variables, various algorithms have been used to develop forest carbon storage models for Ordos. Overall, the machine learning models performed well in estimating carbon storage using Sentinel 2A imagery and the dominant species and types. For Landsat 8 imagery and the entire forest, approach 2 demonstrated higher estimation accuracy than approach 1.

The analysis of forest carbon storage in Ordos showed that although shrubs have a lower carbon density, their extensive coverage results in carbon storage, accounting for 66.52% of the total carbon storage. *Populus* trees accounted for 17.38% of the trees, whereas *Salix*, *P. tabuliformis*, and other species and types accounted for 4.62%, 3.18%, and 8.30%, respectively. Estimations of forest carbon storage for 2013 and 2023 reveal that the total carbon storage in 2023 is 26.74 Mt, an increase of 89.02% compared to 2013.

## Supplementary Information

Supplementary Information.

## Data Availability

The datasets generated and/or analyzed in this study are available from the corresponding author upon reasonable request. Please direct any data access inquiries to liudw@imu.edu.cn.
