# Supplementary material for: Using Sentinel 2A and Landsat 8 imagery to assess changes in forest carbon storage
Source: Sci Rep. 2025 Oct 27;15:37489. doi: 10.1038/s41598-025-21607-0 (PMC12559292; doi:10.1038/s41598-025-21607-0)
Supplement: Supplementary file 1 — Supplementary Information. [file 41598_2025_21607_MOESM1_ESM.docx]

**Satellite Image Data Sources and Processing**

**1. Landsat 8 Data Acquisition and Processing**

Landsat 8 satellite imagery was sourced from the Google Earth Engine platform (<https://earthengine.google.com/>). Two temporal datasets of Landsat 8 OLI imagery were acquired for this study: May-October 2013 and June-September 2023, facilitating carbon density modeling and decadal carbon stock change analysis (Table 1). To maintain remote sensing data quality and usability, high-quality imagery was selected during peak growing seasons with cloud coverage below 5% and minimal extensive shadowing (Table 3).

Image pre-processing was performed using ENVI 5.3 software, encompassing: (1) terrain correction utilizing SRTM DEM data to mitigate geometric distortions induced by topographic variations; (2) radiometric calibration converting digital numbers (DN) to at-sensor radiance; (3) atmospheric correction employing the FLAASH atmospheric correction algorithm to derive surface reflectance products. Subsequently, multiple remote sensing variables were computed to support carbon stock estimation model construction and retrieval analysis.

Table 1 Main parameter information of Landsat 8 OLI satellite.

| Band Number | Band Name | Wavelength Range (μm) | Central Wavelength (μm) | Spatial Resolution (m) |
| --- | --- | --- | --- | --- |
| B1 | Coastal/Aerosol | 0.433-0.453 | 0.443 | 30 |
| B2 | Blue | 0.450-0.515 | 0.482 | 30 |
| B3 | Green | 0.525-0.600 | 0.562 | 30 |
| B4 | Red | 0.630-0.680 | 0.655 | 30 |
| B5 | Near Infrared (NIR) | 0.845-0.885 | 0.865 | 30 |
| B6 | Shortwave Infrared 1 | 1.560-1.660 | 1.610 | 30 |
| B7 | Shortwave Infrared 2 | 2.100-2.300 | 2.200 | 30 |
| B8 | Panchromatic | 0.500-0.680 | 0.590 | 15 |
| B9 | Cirrus | 1.360-1.390 | 1.375 | 30 |
| B10 | Thermal Infrared 1 | 10.30-11.30 | 10.800 | 100 |
| B11 | Thermal Infrared 2 | 11.50-12.50 | 12.000 | 100 |

**2. Sentinel 2A Data Acquisition and Processing**

Sentinel 2A satellite imagery was obtained through the Google Earth Engine platform (<https://earthengine.google.com/>). The study employed Sentinel 2A Level 2A (L2A) products, which are pre-processed with systematic radiometric and atmospheric corrections (Table 2). To maintain temporal alignment between remote sensing acquisitions and field measurements, imagery from the peak growing season corresponding to the field sampling period (July-October 2023) was selected, screening for high-quality images with less than 5% cloud cover and without extensive shadowing (Table 3). Image preprocessing procedures encompassed: spectral band resampling via SNAP software to unify all bands to 10-meter spatial resolution, ensuring spatial consistency across datasets and computing vegetation indices and remote sensing feature variables essential for the research objectives.

Table 2 Main parameter information of Sentinel 2A satellite.

| Band Number | Band Name | Wavelength Range (μm) | Central Wavelength (μm) | Spatial Resolution (m) |
| --- | --- | --- | --- | --- |
| B1 | Coastal Aerosol | 0.430-0.457 | 0.443 | 60 |
| B2 | Blue | 0.440-0.538 | 0.490 | 10 |
| B3 | Green | 0.537-0.582 | 0.560 | 10 |
| B4 | Red | 0.646-0.684 | 0.667 | 10 |
| B5 | Vegetation Red Edge 1 | 0.694-0.713 | 0.705 | 20 |
| B6 | Vegetation Red Edge 2 | 0.731-0.749 | 0.740 | 20 |
| B7 | Vegetation Red Edge 3 | 0.769-0.797 | 0.783 | 20 |
| B8 | Near Infrared | 0.760-0.908 | 0.842 | 10 |
| B8A | Narrow NIR | 0.848-0.881 | 0.865 | 20 |
| B9 | Water Vapor | 0.932-0.958 | 0.945 | 60 |
| B10 | Cirrus | 1.337-1.412 | 1.375 | 60 |
| B11 | Shortwave Infrared 1 | 1.539-1.682 | 1.610 | 20 |
| B12 | Shortwave Infrared 2 | 2.078-2.320 | 2.190 | 20 |

Table 3 Available image information.

| Year | Platform | Name | Time | Cloud Cover |
| --- | --- | --- | --- | --- |
| 2013 | Landsat8 OLI | LC81260332013249LGN01 | 2013/09/06 | 0.15 |
|  |  | LC81270322013256LGN01 | 2013/09/13 | 1.12 |
|  |  | LC81270332013256LGN01 | 2013/09/13 | 0.01 |
|  |  | LC81280322013215LGN01 | 2013/08/03 | 0.07 |
|  |  | LC81280332013215LGN01 | 2013/08/03 | 0.24 |
|  |  | LC81280342013215LGN01 | 2013/08/03 | 0.78 |
|  |  | LC81290322013158LGN01 | 2013/06/07 | 0.49 |
|  |  | LC81290332013158LGN01 | 2013/06/07 | 0.98 |
| 2023 | Landsat8 OLI | LC91260332023205LGN00 | 2023/07/24 | 0.26 |
|  |  | LC91270332023228LGN00 | 2023/08/16 | 0.29 |
|  |  | LC81270322023220LGN00 | 2023/08/08 | 0.09 |
|  |  | LC81280322023227LGN00 | 2023/08/15 | 3.37 |
|  |  | LC91280332023219LGN00 | 2023/08/07 | 0.80 |
|  |  | LC91280342023203LGN01 | 2023/07/22 | 1.31 |
|  |  | LC91290322023242LGN00 | 2023/08/30 | 0.01 |
|  |  | LC91290332023242LGN00 | 2023/08/30 | 0.01 |
|  |  | N0509_R018_T49SCC | 2023/08/21 | 0.02 |
|  |  | N0509_R018_T49SCD | 2023/08/21 | 0.43 |
| 2023 | Sentinel 2A | N0509_R018_T49SDD | 2023/08/21 | 0.02 |
|  |  | N0509_R018_T49TDE | 2023/08/21 | 0.01 |
|  |  | N0509_R061_T48SYH | 2023/09/03 | 0.22 |
|  |  | N0509_R061_T48TYK | 2023/09/03 | 0.01 |
|  |  | N0509_R061_T49SBC | 2023/09/03 | 0.03 |
|  |  | N0509_R061_T49TBE | 2023/09/03 | 0.09 |
|  |  | N0509_R061_T48SXH | 2023/09/13 | 0.01 |
|  |  | N0509_R061_T48SXJ | 2023/09/13 | 0.07 |
|  |  | N0509_R061_T48SYJ | 2023/09/13 | 1.36 |
|  |  | N0509_R061_T48TXK | 2023/09/13 | 0.02 |
| 2023 | Sentinel 2A | N0509_R061_T49SBB | 2023/09/13 | 0.02 |
|  |  | N0509_R061_T49SBD | 2023/09/13 | 0.01 |
|  |  | N0509_R061_T49TBF | 2023/09/13 | 0.28 |
|  |  | N0509_R104_T48TXL | 2023/09/26 | 0.77 |
|  |  | N0509_R018_T49TCE | 2023/09/30 | 0.05 |
|  |  | N0509_R018_T49TEE | 2023/09/30 | 0.09 |
|  |  | N0509_R061_T49TCE | 2023/08/09 | 0.11 |
|  |  | N0509_R018_T49SED | 2023/08/16 | 0.03 |
